# Choroidal neovascularization secondary to tubulointerstitial nephritis and uveitis syndrome (TINU) in an adult patient

**DOI:** 10.1186/s12348-015-0059-7

**Published:** 2015-10-07

**Authors:** Hans Barron Heymann, Daniel Colon, Manjot K. Gill

**Affiliations:** Department of Ophthalmology, Northwestern University Feinberg School of Medicine, 645 N. Michigan Ave, Suite 440, Chicago, IL 60611 USA

## Abstract

**Background:**

Inflammation is a well-known risk factor for the development of choroidal neovascularization (CNV), yet not all causes of intraocular inflammation have been documented to cause CNV. Tubulointerstitial nephritis and uveitis syndrome (TINU) is a rare cause of intraocular inflammation mostly in pediatric patients and only seldom has been associated with development of CNV.

**Findings:**

A 34-year-old pregnant female with a past history of bilateral ocular inflammation secondary to TINU presents 1 year after diagnosis with vision loss in the left eye. Clinical examination and investigations show the development of CNV in the left eye. The patient was treated with ranibizumab (Genentech, San Francisco, CA) intravitreal injections with improvement in symptoms and clinical findings.

**Conclusions:**

We report the first case of CNV secondary to TINU in an adult patient. The CNV associated with TINU is responsive to intravitreal anti-vascular endothelial growth factor (anti-VEGF) therapy.

## Findings

### Introduction

Various conditions that lead to intraocular inflammation can be associated with the development of choroidal neovascularization (CNV) [1, 2]. Tubulointerstitial nephritis and uveitis syndrome (TINU) is a rare entity first described in 1975 by Dobrin and colleagues [3], and has seldom been associated with secondary CNV [4]. Since TINU was first described, there have been more than 150 cases reported mostly in the pediatric nephrology and ophthalmology literature [5, 6]. To date, however, there have been only two documented cases of CNV secondary to TINU reported in the literature [4] with both of these cases in pediatric patients. Herein we report a case of CNV secondary to TINU in an adult patient which to our knowledge is the first such to be described.

### Case description

A 34-year-old pregnant female at 33 weeks gestation presented as a consult to the retina service with chief complaint of sudden decreased vision in the left eye beginning 1 month ago. The patient was first diagnosed with TINU 1 year prior by a uveitis specialist. At that time, she had presented with an inflamed, painful left eye, 2+ cell and flare, posterior synechiae, and a negative work up—including rapid plasma reagin, quantiFERON Gold test, FTA-ABS, human leukocyte antigen (HLA)-B27 analysis by flow cytometry, and Lyme titers. Serum beta 2-microglobulin was found to be 2.43 (normal <1.80 mg/dl), and urine beta 2-microglobuin was found to be 4.70 mg/dl (normal <0.24 mg/dl). Based on the clinical picture and laboratory findings, a diagnosis of TINU was made and the patient was referred to nephrology for renal evaluation. The patient was subsequently lost to follow-up.

**Fig. 1:**
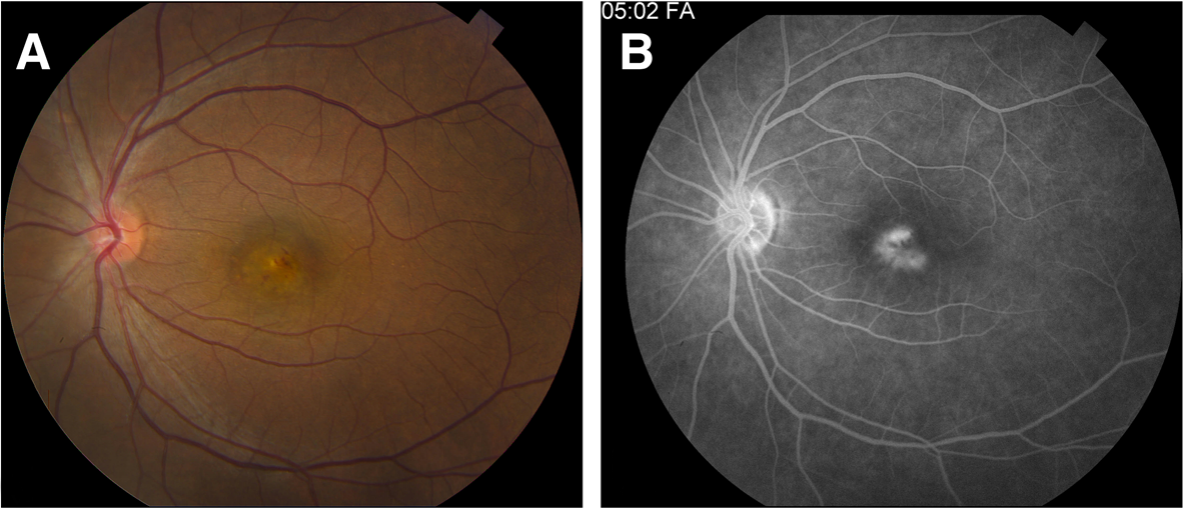
**a** Color fundus photo of left eye shows pinpoint hemorrhage overlying the fovea. **b** Fluorescein angiography of left eye demonstrates late phase parafoveal leakage consistent with neovascularization

**Fig. 2:**
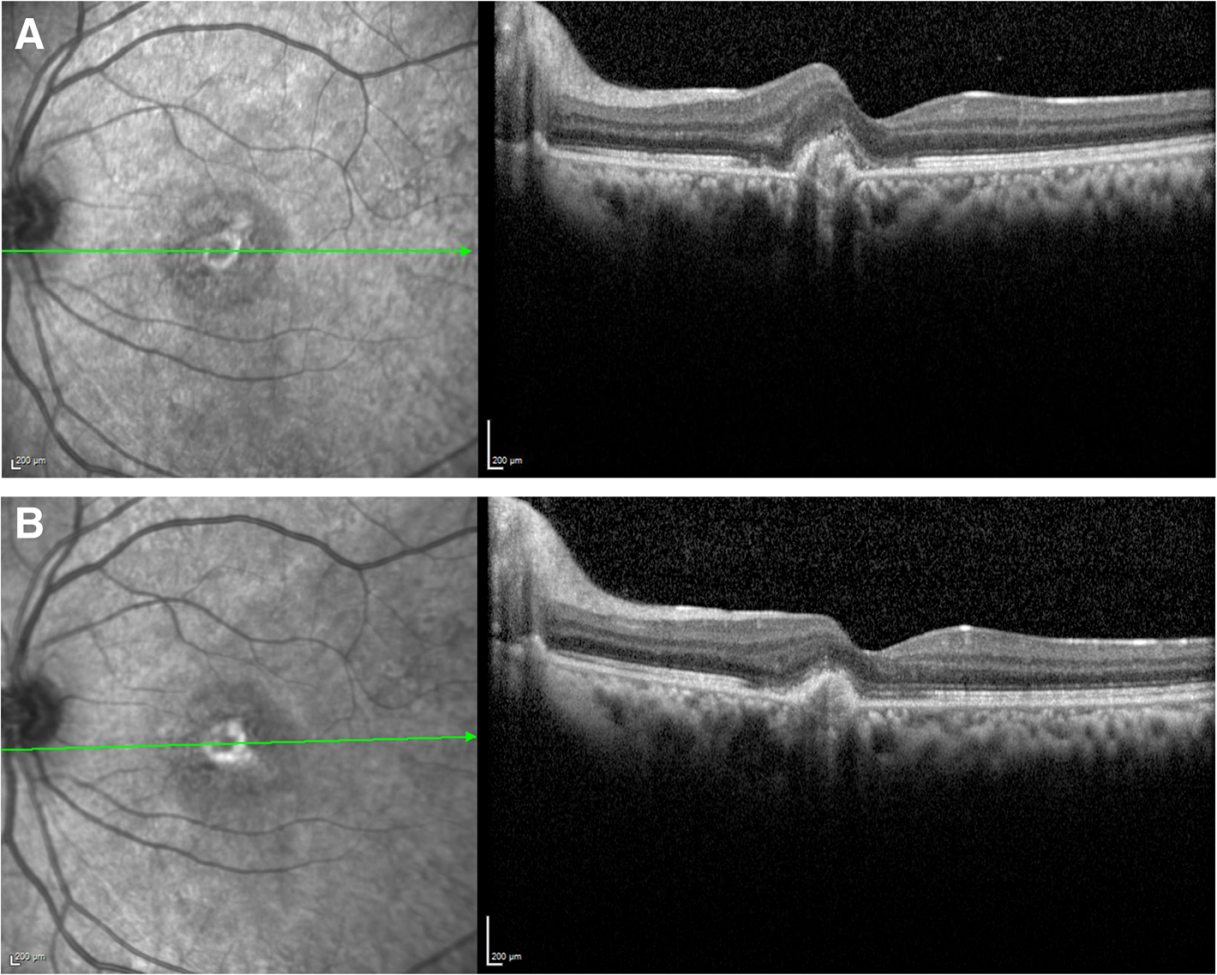
**a** SD-OCT image through fovea reveals disruption of Bruch’s membrane and overlying photoreceptor layer. **b** SD-OCT image demonstrating consolidation of choroidal neovascularization post-treatment with ranibizumab

On her presentation to the retina service, the patient’s best corrected visual acuity was found to be 20/500 in her left eye. The anterior segment examination was significant for pigment on the anterior capsule of the lenses bilaterally consistent with prior inflammation. There was no active intraocular inflammation in either eye. Her dilated fundus exam of the left eye was significant for central thickening of the macula with pinpoint parafoveal hemorrhage (Fig. 1a). Spectral-domain optical coherence tomography (SD-OCT) imaging showed distortion of foveal contour and a break in Bruch’s membrane with disruption of overlying photoreceptors consistent with choroidal neovascularization (Fig. 2a). Fluorescein angiography was deferred as the patient was pregnant. To support further the diagnosis of TINU, the patient was tested for HLA-DRB1*0102 and the result was positive for subtype DR1,8; DQ 4,5. The patient was offered intraocular anti-vascular endothelial growth factor (anti-VEGF) therapy or intraocular steroids; however, following discussion of the risks, benefits, and alternatives, the patient declined any treatment until after delivery.

The patient delivered at 40 weeks gestation and was seen 3 days post-partum. The patient’s visual acuity, exam, and SD-OCT were unchanged. Fluorescein angiography was obtained which revealed leakage consistent with a diagnosis of CNV (Fig. 1b). The patient was not breastfeeding and was treated with two intravitreal injections of ranibizumab at 1 month apart. At the most recent follow-up 8 weeks following the first injection, BCVA improved to 20/50 in the left eye. OCT imaging showed consolidation of the choroidal neovascularization (Fig. 2b).

### Discussion

Tubulointerstitial nephritis and uveitis is an uncommon entity that is described mostly in children with a median age of diagnosis of 15 years and has a 3:1 female predominance [7, 8]. Establishing the diagnosis of TINU is challenging and requires clinical suspicion. No specific laboratory study is diagnostic of TINU, but there are several laboratory abnormalities that suggest the diagnosis in the proper clinical context. Patients with TINU have been reported to have anemia, eosinophilia, elevations in ESR and CRP, sterile pyuria and abnormal elevations in serum, and urine beta-2 microglobulin [9, 10]. Renal disease can be confirmed by biopsy, which shows tubulointerstitial edema and infiltration of lymphocytes, plasma cells and histiocytes, but this is only rarely done [11]. More recently, a strong association has been shown between TINU and the HLA-DRB1*0102 [10]. The diagnosis of TINU was made clinically in our patient with demonstration of bilateral anterior uveitis, elevated serum and urine beta-2 microglobulin, and otherwise negative workup for other causes of uveitis. As an additional measure, we tested the patient for HLA-DRB1*0102. The positive result strongly supports the clinical diagnosis of TINU.

Generally, the ocular inflammation of TINU preferentially affects the anterior segment, although posterior inflammation has been reported [4–8]. Intraocular inflammation is a well-established risk factor for the development of CNV and has been described in association with a wide variety of inflammatory conditions including multifocal choroiditis and uveitis syndrome, punctate inner choroidopathy, and ocular histoplasmosis [1, 2]. Rarely has CNV been described secondary to TINU and only in the pediatric population [4]. Our patient clearly demonstrated SD-OCT and angiographic evidence of choroidal neovascularization in the absence of other identifiable causes. This is the first reported case of CNV secondary to TINU in an adult, and as such, patients should be counseled and monitored for the occurrence of this rare complication.

Multiple studies have demonstrated the efficacy of anti-VEGF therapy in the treatment of CNV due to age-related macular degeneration [12, 13]. There are, however, no established guidelines for treatment of inflammatory CNV. The small number of patients with these rare conditions preclude the possibility of large randomized trials to evaluate treatment efficacy. A recent review, however, evaluated 16 studies of anti-VEGF therapy for CNV secondary to non-age-related macular degeneration conditions and concluded that positive treatment outcomes in these patients should encourage clinicians to consider bevacizumab and ranibizumab for rare causes of CNV [14]. We conclude based on these considerations, and the favorable outcome for our patient, that treatment with anti-VEGF should be considered as these lesions appear to show a favorable response to treatment.

## Consent to publish

Consent to publish relevant clinical photographs has been obtained from the patient involved in this case.

## Abbreviations

CNV: choroidal neovascularization
HLA: human leukocyte antigen
SD-OCT: spectral-domain optical coherence tomography
TINU: tubulointerstitial nephritis and uveitis syndrome
